# Textual analysis of 38 Chinese subnational tobacco control regulations against FCTC Article 8

**DOI:** 10.18332/tid/222367

**Published:** 2026-06-12

**Authors:** Yu Chen, Xinjie Zhao, Chunyan Chen, Xinyi Zhang, Jinghan Li, Jiayi Lan, Shiyu Liu, Xinyao Yu, Kin-Sun Chan, Qian Zeng

**Affiliations:** 1 School of Art and Communication, Fujian Polytechnic Normal University, Fuqing, China; 2 School of Journalism and Communication, Peking University, Beijing, China; 3 School of Public Health, Xi’an Jiaotong University, Xi'an, China; 4 Faculty of Social Sciences, University of Macau, Macau, China; 5 Northwest University of Political Science and Law, Xi'an, China

**Keywords:** FCTC Article 8, tobacco control legislation, China, subnational law, text analysis

## Abstract

**INTRODUCTION:**

China ratified the WHO Framework Convention on Tobacco Control (FCTC) in 2005; it entered into force domestically on 9 January 2006. Absent of comprehensive national smoke-free legislation, subnational jurisdictions have enacted dedicated tobacco control regulations heterogeneously. The textual architecture distinguishing FCTC Article 8-compliant from non-compliant drafting has not been systematically characterized at corpus scale.

**METHODS:**

This study compiled 38 Chinese subnational dedicated tobacco control regulations enacted or materially amended after FCTC entry into force (9 January 2006 through 31 December 2024) (100635 substantive Chinese characters). Each was independently evaluated against the four core requirements of the FCTC Article 8 Implementation Guidelines [FCTC/COP2(7), 2007] and triangulated against an authoritative expert database. A five-layer framework examined corpus scale, compliance, e-cigarette prohibitions, enforcement features, and other FCTC complementary measures. Mann–Whitney U tests with Cliff’s delta and Fisher’s exact tests were used (two-sided, α = 0.05).

**RESULTS:**

Of 38 regulations, 10 (26.3%) met FCTC Article 8 compliance criteria; 28 (73.7%) did not. Compliant regulations had higher median character counts (3249 vs 2572; δ=0.40, p=0.066) and clause counts (28.0 vs 23.0; δ=0.46, p=0.034). E-cigarette prohibitions (60.0% vs 25.0%, p=0.062) and cessation service requirements (90.0% vs 50.0%, p=0.056) were more frequent in compliant regulations. The 10 compliant jurisdictions cover an estimated 121.6 million residents (8.6% of mainland China’s 2020 population).

**CONCLUSIONS:**

Greater corpus elaboration, more complete enforcement specification, and inclusion of FCTC-aligned complementary measures are textual features systematically associated with – though not shown to cause – FCTC Article 8 compliance in Chinese subnational law. These findings characterize legal text rather than implementation outcomes and inform drafting guidance for Chinese cities and other jurisdictions pursuing Article 8 implementation.

## INTRODUCTION

Tobacco use remains the single largest preventable cause of premature death globally, with China accounting for approximately 300 million smokers and nearly 40% of worldwide cigarette consumption^1^. China ratified the World Health Organization Framework Convention on Tobacco Control (FCTC) in 2005, and the treaty entered into force domestically on 9 January 2006^1^. Article 8 of the Convention obligates Parties to adopt and implement effective measures providing protection from exposure to tobacco smoke in indoor workplaces, public transport, indoor public places, and, as appropriate, other public places; the 2007 implementation guidelines specify that only complete elimination of smoking in enclosed indoor environments, with no designated smoking areas or ventilation-based exceptions, provides adequate protection^2^. Nearly two decades after ratification, China has yet to enact comprehensive national smoke-free legislation, and FCTC Article 8 implementation has proceeded through subnational pathways^3-5^.

This study draws on insights from legal epidemiology, regulatory design, and policy implementation research to examine how legal text features may relate to health-protective outcomes^6^. Legal epidemiology emphasizes that the wording, scope, and enforcement architecture embedded in statutes themselves shape the protection laws they can deliver, independent of implementation context. Within this perspective, corpus-level analysis of regulatory text – examining how legal instruments are constructed at scale – offers a complementary lens to outcome-based evaluation and policy-process research.

Prior analyses of Chinese subnational tobacco control legislation have established two important baselines. Lin et al.^5^ assessed 21 city-level smoke-free laws against FCTC Article 8 requirements using a legal-compliance checklist across 125 cities, identifying provincial-capital status as the sole statistically significant adoption predictor. A recent China CDC Weekly analysis expanded the inventory to 140 regulations spanning dedicated tobacco control, public health, and civil-behavior instruments, demonstrating that 46 cities achieve complete smoking bans across all eight WHO public-place categories and quantifying population coverage at 238.76 million^4^. The Health Law Research Center of China University of Political Science and Law, with technical support from Caixin Data Visualization Lab, maintains an authoritative dynamic database classifying Chinese subnational jurisdictions against FCTC Article 8 criteria^7^. Yang et al.^8^ complemented these baselines with qualitative analysis of the diversity and impacting factors of smoke-free legislation in mainland China. These prior efforts answer what is regulated where and which jurisdictions achieve compliance at a given point in time; none characterizes the textual architecture that distinguishes compliant from non-compliant drafting at corpus scale, nor systematically compares coded features across the full subnational dedicated corpus.

This study addresses this gap through the first systematic textual analysis of the full Chinese-character corpus of subnational dedicated tobacco control regulations enacted or materially amended after China’s FCTC entry into force on 9 January 2006 through 31 December 2024. The objectives are threefold: 1) to document the corpus-level textual landscape that distinguishes compliant from non-compliant drafting; 2) to identify the enforcement and complementary-measure features that are most strongly associated with compliance; and 3) to inform drafting and maintenance guidance applicable to future subnational tobacco control legislation in China and, by extension, comparable jurisdictions.

### Operational definitions of key analytical terms

*Textual architecture*

The configuration of corpus-level features of a regulatory text, encompassing character count, clause count, enforcement infrastructure (penalty schedules, enforcement authority, complaint mechanisms), and the presence of FCTC-aligned complementary provisions. The term denotes the way a regulation is constructed as a text, not the legal effects it produces.

*Corpus scale*

The total character and clause count of a regulation, providing a measure of drafting elaboration independent of substantive content.

*Character count*

The number of Chinese characters contained in the substantive clause text of a regulation, comprising the numbered articles that establish prohibitions, definitions, enforcement authority, penalties, and complementary provisions. Preambles, signature blocks, structural headers (chapter and section titles), and dated promulgation metadata are excluded; chapter-internal definition articles and effective-date articles, where placed as numbered articles, are included.

*Clause count*

The number of numbered articles in the regulation. Subsections and items within an article are not counted separately.

*Compliance criteria*

The four core criteria of the FCTC Article 8 Implementation Guidelines are: 1) Effectively comprehensive coverage of indoor public places, indoor workplaces, and public transport; 2) No buffer period; 3) No designated smoking rooms or smoking areas; and 4) Clearly specified enforcement authority and penalty schedules.

*Material amendment*

An amendment that altered substantive provisions such as scope of covered places, exception categories, enforcement authority, or penalty levels, as distinct from purely editorial or technical amendments.

## METHODS

### Corpus construction and search strategy

This study systematically assembled the corpus of all dedicated subnational tobacco control regulations enacted in mainland China in the period following the FCTC’s entry into force on 9 January 2006 through 31 December 2024. Three complementary sources were searched between 1 February and 30 April 2025: 1) the National Database of Laws and Regulations; 2) the PKULaw database; and 3) provincial and municipal People’s Congress and People’s Government official websites for each provincial-level unit in mainland China.

Search terms used included Chinese-language combinations equivalent to ‘smoking control’, ‘smoking prohibition’, ‘public places’, ‘secondhand smoke’, and the instrument-type terms regulation, provision, and measure. The search strategy was anchored on smoke-free terminology as the core legal hook in Chinese subnational tobacco control drafting, in correspondence with FCTC Article 8 – the focal Convention article of this study. Other tobacco control measures co-occurring within these dedicated instruments (advertising restrictions, cessation services, minor protection, and health education) are captured as distinct coding dimensions in Layers 3 and 5 of the analytical framework rather than as separate search anchors, since these complementary measures typically appear as additional provisions inside the smoke-free instruments themselves. Records were screened against three inclusion criteria: 1) primarily concerned with tobacco or smoking control rather than embedded within public health, civil-behavior, or other broader regulations; 2) formally enacted and in force as of 31 December 2024; and 3) issued at the provincial or prefecture-level-city tier. Duplicates across databases were identified by exact title match and consolidated to the most recent in-force version. The full document selection pathway is presented in Supplementary file Figure S1.

Chinese subnational legal instruments span a hierarchy from comprehensive local regulations issued by People’s Congresses through government regulations issued by People’s Governments to supplementary administrative rules issued by government offices. Public health regulations and civil-behavior regulations, which sometimes contain smoke-free provisions, sit alongside dedicated tobacco control instruments at the same hierarchical level but address broader subject matter. The decision to exclude embedded provisions from the analytical corpus preserves textual homogeneity for corpus-level comparison but necessarily restricts the scope of findings to dedicated instruments; this scope decision is acknowledged among the limitations.

**Figure 1:**
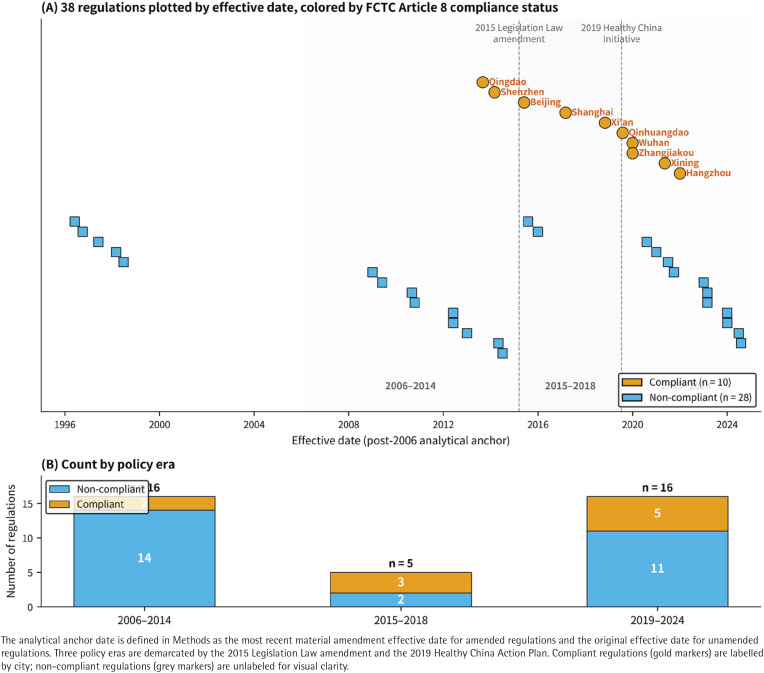
Temporal distribution of 38 Chinese subnational dedicated tobacco control regulations enacted or materially amended after FCTC entry into force on 9 January 2006 through 31 December 2024, by analytical anchor date (N=38; compliant N=10; non-compliant N=28)

For regulations originally enacted before 9 January 2006 but materially amended thereafter, the most recent in-force text was analyzed. Two temporal variables were used throughout: 1) the original effective date of the regulation as initially promulgated, retained in Table 1 for historical reference; and 2) the analytical anchor date, defined as the most recent material amendment effective date for amended regulations and the original effective date for unamended regulations. All temporal analyses, including Figure 1, use the analytical anchor date. The final corpus comprises 38 regulations totaling 100635 Chinese characters of substantive clause text.

**Table 1:** Inventory of 38 Chinese subnational dedicated tobacco control regulations enacted or materially amended after FCTC entry into force on 9 January 2006 through 31 December 2024

ID	Regulation (city)	Province	Level	Effective	Amended	Art.	Chars	FCTC
1	Anyang Public Places Smoking Control Regulation	Henan	GR	2024-06-28	-	19	1910	✗
2	Shangqiu Public Places Smoking Control Measures	Henan	AM	2023-12-29	-	22	2494	✗
3	Suzhou (Anhui) Public Places Smoking Control Regulation	Anhui	LR	2024-08-01	-	12	1602	✗
4	Zhuhai SEZ Public Places Smoking Control Regulation	Guangdong	LR	2024-01-01	-	26	3594	✗
5	Hangzhou Public Places Smoking Control Regulation	Zhejiang	LR	2022-01-01	-	35	3885	✓
6	Shenzhen SEZ Smoking Control Regulation	Guangdong	LR	2014-03-01	2019-06-26	46	5357	✓
7	Fuzhou Public Places Smoking Control Regulation	Fujian	LR	2015-08-01	2015-05-28	21	2649	✗
8	Changchun Tobacco Smoke Hazards Prevention Measures	Jilin	AM	2014-03-01	-	31	2882	✗
9	Nanning Smoking Control Regulation	Guangxi	LR	2014-07-01	-	27	3174	✗
10	Tangshan Second-hand Smoke Prevention Management Measures	Hebei	AM	2014-05-01	-	26	2708	✗
11	Qingdao Smoking Control Regulation	Shandong	LR	2013-09-01	-	16	2537	✓
12	Anshan Public Places Smoking Control Regulation	Liaoning	GR	2013-01-01	-	18	2458	✗
13	Tianjin Smoking Control Regulation	Tianjin	LR	2012-05-31	-	27	3638	✗
14	Shijiazhuang Public Places Smoking Prohibition Regulation	Hebei	GR	2010-10-14	2010-08-26	14	1355	✗
15	Jinan Public Places Smoking Prohibition Regulation	Shandong	GR	1996-05-31	2010-10-27	15	1625	✗
16	Hohhot Public Places Smoking Prohibition Regulation	Inner Mongolia	GR	2009-01-01	2008-11-04	12	1231	✗
17	Yinchuan Public Places Smoking Control Regulation	Ningxia	LR	2009-06-01	-	15	1639	✗
18	Urumqi Public Places Smoking Prohibition Regulation	Xinjiang	GR	1998-03-01	2010-06-24	14	1333	✗
19	Luoyang Public Places Smoking Prohibition Regulation	Henan	GR	1996-10-01	2010-11-12	14	1232	✗
20	Beijing Smoking Control Regulation	Beijing	LR	2015-06-01	2021-09-24	30	3337	✓
21	Chongqing Public Places Smoking Control Regulation	Chongqing	LR	2021-01-01	-	25	3929	✗
22	Lanzhou Public Places Smoking Control Regulation †	Gansu	LR	2016-01-01	2018-12-01	29	3350	✗
23	Shenyang Smoking Control Regulation	Liaoning	LR	2021-10-01	-	28	3057	✗
24	Dalian Smoking Control Regulation	Liaoning	LR	2021-07-01	-	28	3105	✗
25	Xining Smoking Control Regulation	Qinghai	LR	2021-05-12	2021-03-31	23	2543	✓
26	Qinhuangdao Smoking Control Measures	Hebei	AM	2019-08-01	2021-01-25	26	3161	✓
27	Datong Public Places Smoking Prohibition Regulation	Shanxi	GR	1997-05-31	2021-01-15	13	1288	✗
28	Zhengzhou Public Places Smoking Prohibition Regulation	Henan	LR	2020-08-05	2020-08-05	17	1756	✗
29	Wuhan Smoking Control Regulation	Hubei	LR	2020-01-01	-	36	3677	✓
30	Harbin Second-hand Smoke Hazards Prevention Regulation	Heilongjiang	LR	2012-05-31	2020-06-30	28	3136	✗
31	Xi’an Tobacco Control Administrative Measures	Shaanxi	AM	2018-11-01	2020-12-31	31	3456	✓
32	Guangzhou Smoking Control Regulation	Guangdong	LR	2010-09-01	2020-07-29	31	3957	✗
33	Zhangjiakou Public Places Smoking Control Regulation	Hebei	LR	2020-01-01	-	21	1790	✓
34	Guiyang Public Places Smoking Prohibition Provisional Regulation	Guizhou	GR	1998-06-24	2022-12-19	9	780	✗
35	Chengdu Public Places Smoking Control Regulation	Sichuan	LR	2023-01-01	-	25	3617	✗
36	Weihai Smoking Control Management Measures	Shandong	AM	2023-03-01	-	27	2844	✗
37	Weifang Smoking Control Management Measures	Shandong	AM	2023-03-01	-	24	2208	✗
38	Shanghai Public Places Smoking Control Regulation	Shanghai	LR	2017-03-01	2022-10-28	23	2341	✓

LR: local regulation (issued by a People’s Congress). GR: government regulation (issued by a People’s Government). AM: administrative measure. Effective: original effective date. Amendment: most recent material amendment effective date. FCTC Article 8: ✓ compliant under independent evaluation; ✗ non-compliant. The gradient of regulatory protection within the non-compliant category is characterized through the granular coded variables reported in Table 3.

† Lanzhou: parent ordinance dates only; the 2018 Implementing Rules (effective 1 December 2018 – 30 November 2023) lifted Lanzhou to compliance for five years before expiring unrenewed, after which the permissive parent Article 10 reverted to controlling force (see Methods and Discussion). Source documents were retrieved from the National Database of Laws and Regulations, the PKULaw database, and provincial/municipal People’s Congress and People’s Government websites (accessed 1 February – 30 April 2025).

### FCTC Article 8 compliance evaluation

Each regulation was independently evaluated against the four core requirements specified in the FCTC Article 8 Implementation Guidelines adopted at the Second Session of the Conference of the Parties [FCTC/COP2(7), 2007]^2^ : 1) effectively comprehensive coverage of indoor public places, indoor workplaces, and public transport – operationalized as either a generic three-venue formulation or an enumerative list that substantively covers all three categories without exceptions for hospitality, entertainment, or workplace venues; 2) no buffer period permitting delayed implementation in specific venue categories; 3) no designated smoking rooms, smoking areas, or ventilation-based exceptions in the regulatory text; and 4) clearly specified enforcement authority and penalty schedules. A regulation was classified as FCTC Article 8-compliant only when all four requirements were met.

Two researchers independently coded all 38 regulations against this binary criterion; inter-coder agreement was high (Cohen’s κ=0.94)^9^. Disagreements were resolved by discussion with reference to the original legislative text. Worked examples illustrating coding decisions for ‘effectively comprehensive coverage’ versus enumerative listings with gaps are provided in the Supplementary file Box S1. The binary compliant/non-compliant classification follows the conjunctive logic of the FCTC Article 8 Implementation Guidelines themselves – a regulation either satisfies all four core requirements or it does not – and is therefore directly comparable with prior compliance assessments using the same framework^2-5^. To characterize the gradient of regulatory protection within the non-compliant category, the granular coded variables in Table 3 are reported separately rather than reduced to a single summary attribution.

Compliance classification was based on the main ordinance text as the primary legal instrument; where an implementing rule was in force at the coding date and materially modified the operational scope, its content was evaluated separately and its effect on compliance status was documented. This methodological choice proved consequential for Lanzhou, whose 2018 implementing rules had lifted the main ordinance to substantive Article 8 compliance for five years before lapsing unrenewed on 30 November 2023^10^. As of the analytical cutoff date (31 December 2024), no extension notice or transitional rule had been promulgated in PKULaw or the National Database of Laws and Regulations. The independent classification was triangulated against the parallel classification maintained by the Health Law Research Center of China University of Political Science and Law in the Healthy China Smoke-Free Legislation interactive database^7^. Core classifications converged in all cases; the single divergence concerned Lanzhou, where this study’s evaluation incorporated the November 2023 expiration of the implementing rules and accordingly classified Lanzhou as non-compliant, a status not yet reflected in the public-facing database at the time of analysis.

### Five-layer analytical framework

A five-layer analytical framework was developed iteratively to integrate corpus-level linguistic features with FCTC-aligned coded variables. The framework is inductive in origin but anchored in the structure of the FCTC Article 8 Implementation Guidelines and the conventional architecture of dedicated tobacco control regulations: the first layer addresses corpus-level drafting elaboration; the second layer applies the four core compliance criteria; the third layer isolates e-cigarette prohibitions as a recent FCTC-aligned policy area; the fourth layer examines enforcement-related features (some of which operationalize the compliance criteria themselves); and the fifth layer covers other FCTC complementary measures drawn from Convention articles commonly co-occurring in dedicated tobacco control regulations. A schematic of the framework is presented in Supplementary file Figure S2.

The first layer characterized corpus scale and structure through character counts and clause counts per regulation, providing drafting-elaboration measures without imposing theoretical content assumptions. The second layer presented the FCTC Article 8 compliance classification described above, identifying the cohort of 10 compliant regulations against which the subsequent layers were compared. The third layer examined e-cigarette prohibitions, separated from other complementary measures in recognition of their status as a recent FCTC-aligned policy area following the 2016 COP7 report on electronic nicotine delivery systems^11^ and the 2019 WHO guidance, and the November 2022 State Tobacco Monopoly Administration e-cigarette rules^12^. The fourth layer examined enforcement-related features including individual and venue penalty schedules, enforcement authority specification, and complaint and reporting mechanisms; the first three of these variables are constituent components of compliance criterion 4, and comparisons between compliant and non-compliant regulations on these variables are therefore partly definitional by construction. This relationship is made transparent in Results and the corresponding comparisons are presented as descriptive characterizations of how criterion 4 is operationalized rather than as independent tests of association. Complaint and reporting hotlines are not part of the compliance criteria and serve as an independent variable. The fifth layer examined other FCTC complementary measures drawn from Convention articles on tobacco advertising (Article 13), minor protection (Article 16), cessation services (Article 14), and health education (Article 12).

Broader determinants of effective tobacco control – including governance capacity, enforcement context, and health-system characteristics – lie outside the textual scope of this study and are addressed by complementary outcome-based and implementation studies cited in the Discussion.

### Temporal and statistical analysis

Temporal analysis divided the corpus into three policy eras framed by two national legislative milestones. The 2015 amendment to the Legislation Law extended local legislative authority from the previous 49 larger municipalities and provincial capitals to all 284 prefecture-level cities, producing a structural acceleration of subnational lawmaking from 2015 onward^13^. The 2019 Healthy China Action Plan (Healthy China Initiative) established tobacco control as one of 15 dedicated action areas under State Council oversight, setting a target adult smoking prevalence of 20% by 2030 and intensifying political commitment to subnational legislative action^14^. These two nodes define the three eras used in the analysis: 2006–2014 (pre-Legislation Law amendment), 2015–2018 (post-Legislation Law consolidation), and 2019–2024 (Healthy China Initiative era).

Mann–Whitney U tests (two-sided) with Cliff’s delta effect sizes compared compliant and non-compliant regulations on continuous measures^15^; Fisher’s exact tests compared categorical coded variables^16^. The threshold for statistical significance was set at α=0.05 (two-sided). Given the modest sample size of compliant regulations (n=10), statistical power was limited for detecting differences in low-prevalence variables; p-values near conventional thresholds (e.g. p ≈ 0.05–0.07) are accordingly interpreted as observed trends rather than confirmed differences. All analyses used Python 3.11 (SciPy 1.11, pandas 2.2) and R 4.4.

### Ethics

This study analyzed publicly available legislative documents and did not involve human subjects; thus, ethics approval was not required.

## RESULTS

### Layer 1: Corpus scale and structure

**Figure 2:**
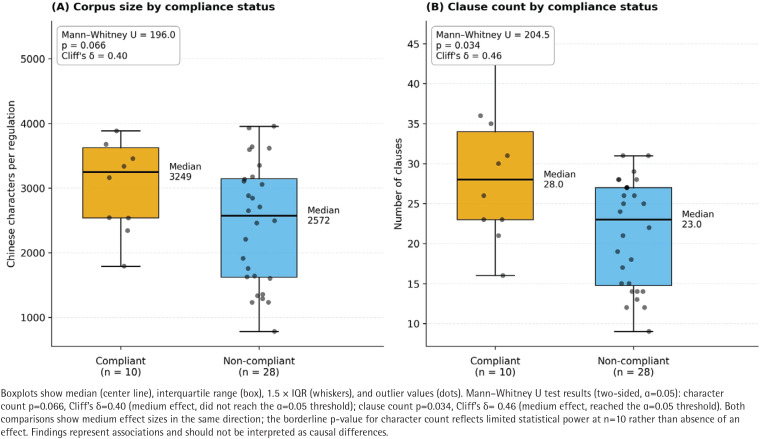
Distribution of character count (top) and clause count (bottom) between FCTC Article 8-compliant and non-compliant regulations, China, 2006–2024 (N=38; compliant N=10; non-compliant N=28)

The final corpus comprised 38 regulations totaling 100635 Chinese characters of substantive clause text, with individual regulations ranging from 780 to 5357 characters (median 2678.5). Median character count was 3249 (IQR: 2538–3622; range: 1790–5357) for compliant regulations compared with 2572 for non-compliant regulations (IQR: 1619–3146; range: 780–3957); Mann–Whitney U=196.0, p=0.066, Cliff’s δ=0.40, representing a medium effect size. Median clause count was 28.0 for compliant regulations (IQR: 23–34; range: 16–46) compared with 23.0 for non-compliant regulations (IQR: 15–27; range: 9–31); Mann–Whitney U=204.5, p=0.034, Cliff’s δ=0.46, also a medium effect size and reaching the conventional α=0.05 significance threshold (Figure 2, Table 2).

**Table 2:** Corpus scale and clause count comparison between FCTC Article 8-compliant and non-compliant regulations, China, 2006–2024 (N=38; compliant N=10; non-compliant N=28)

*Variable*	*Compliant (N=10)*	*Non-compliant (N=28)*	*Mann–Whitney U*	*p*	*Cliff’s δ*	*Effect*
Median character count (IQR; range)	3249 (2538–3622; 1790–5357)	2572 (1619–3146; 780–3957)	196.0	0.066	0.40	Medium
Median clause count (IQR; range)	28.0 (23–34; 16–46)	23.0 (15–27; 9–31)	204.5	0.034*	0.46	Medium

Statistical significance threshold: α=0.05 (two-sided). Cliff’s δ effect size interpretation: |δ| <0.147 negligible; 0.147–0.330 small; 0.330–0.474 medium; ≥ 0.474 large. IQR: interquartile range.

* p<0.05.

**Table 3:** FCTC-aligned coded variables (Layers 3–5) by compliance status, China, 2006–2024 (N=38; compliant N=10; non-compliant N=28)

*Variable*	*Compliant (N=10) n (%)*	*Non-compliant (N=28) n (%)*	*Total (N=38) n (%)*	p*
**Panel A. Layer 3 – Emerging FCTC-aligned area** (independent variable)				
E-cigarette prohibition	6 (60.0)	7 (25.0)	13 (34.2)	0.062
**Panel B. Layer 4 – Enforcement-related features**				
**Components of compliance criterion 4 – comparisons are descriptive by construction** (not independent tests)				
Individual violation penalty specified	10 (100)	25 (89.3)	35 (92.1)	na^†^
Venue violation penalty specified	10 (100)	24 (85.7)	34 (89.5)	na^†^
Enforcement authority specified	10 (100)	22 (78.6)	32 (84.2)	na^†^
**Independent variable** (not part of compliance criteria)				
Complaint/reporting hotline	5 (50.0)	7 (25.0)	12 (31.6)	0.235
**Panel C. Layer 5 – Other FCTC complementary measures** (independent variables)				
Ban on cigarette sales to minors (Art. 16)	8 (80.0)	16 (57.1)	24 (63.2)	0.269
Ban on e-cigarette sales to minors (Art. 16)	1 (10.0)	6 (21.4)	7 (18.4)	0.650
Tobacco advertising restrictions (Art. 13)	6 (60.0)	15 (53.6)	21 (55.3)	1.000
Cessation services (Art. 14)	9 (90.0)	14 (50.0)	23 (60.5)	0.056
Health education provisions (Art. 12)	10 (100)	27 (96.4)	37 (97.4)	1.000

Statistical significance threshold: α=0.05 (two-sided).

* Two-sided Fisher’s exact tests with α=0.05.

† Variables in this subsection are components of compliance criterion 4; all compliant regulations specify them by classification design, so independent tests of association are not meaningful and have been omitted. na: not available.

### Layer 2: FCTC Article 8 compliance classification

Independent evaluation against the four core requirements of the FCTC Article 8 Implementation Guidelines classified 10 of the 38 regulations (26.3%) as FCTC-compliant and 28 (73.7%) as non-compliant (Table 1). The 10 compliant regulations are Qingdao 2013, Shenzhen 2014 (as amended 2019), Beijing 2015 (as amended 2021), Shanghai 2017 (as amended 2022), Xi’an 2018 (as amended 2020), Qinhuangdao 2019 (as amended 2021), Wuhan 2020, Zhangjiakou 2020, Xining 2021, and Hangzhou 2022. Non-compliance arose most commonly from exception clauses permitting designated smoking areas in hospitality venues, followed by enumerative listings of covered places that omitted relevant categories, and spatial qualifiers limiting the ban to ‘urban districts’ narrowly interpreted.

Enactment or amendment clustered in three policy eras tied to national legislative milestones: 15 regulations (39.5%) in the 2006–2014 pre-Legislation Law period, 5 regulations (13.2%) in the 2015–2018 post-Legislation Law consolidation period, and 18 regulations (47.4%) in the 2019–2024 Healthy China Initiative period (Figure 1). Compliant regulations were distributed across all three policy eras, with the post-2019 cohort representing half of all compliant regulations. The independent classification converged with the authoritative expert classification maintained by the Health Law Research Center of China University of Political Science and Law; the single divergence concerned Lanzhou, examined separately below.

### Layer 3: E-cigarette prohibitions as a recent FCTC-aligned policy area

Thirteen regulations (34.2% of the corpus) contain explicit e-cigarette prohibition clauses (Table 3). The proportion was higher among compliant regulations (6/10; 60.0%) than non-compliant regulations (7/28; 25.0%); Fisher’s exact p=0.062, an observed trend that did not reach the α=0.05 threshold. Shenzhen 2014 was the first regulation in the corpus to include an e-cigarette prohibition. Subsequent adoption accelerated after 2020: 8 of the 13 e-cigarette-inclusive regulations were enacted from 2020 onward.

### Layer 4: Enforcement-related features

Components of compliance criterion 4 – individual violation fines, venue violation fines, and explicit identification of enforcement authority – were specified in all 10 compliant regulations (100%) by classification design, compared with rates of 89.3%, 85.7%, and 78.6%, respectively, among non-compliant regulations (Table 3). These three comparisons describe the operational specification of criterion 4 and are not independent tests of association. The variability observed among non-compliant regulations indicates that several of these instruments include some, but not all, components of criterion 4.

Complaint and reporting hotlines – which are not part of the compliance criteria – appeared in 5 of 10 compliant regulations (50.0%) and 7 of 28 non-compliant regulations (25.0%); Fisher’s exact p=0.235. This is the only Layer 4 variable that permits an independent comparison; the difference observed represents an observed trend that did not reach statistical significance at α=0.05.

### Layer 5: Other FCTC complementary measures

Cessation service requirements (FCTC Article 14) appeared in 9 of 10 compliant regulations (90.0%) compared with 14 of 28 non-compliant regulations (50.0%); Fisher’s exact p=0.056, an observed trend that did not reach the α=0.05 threshold. Ban on cigarette sales to minors (FCTC Article 16) appeared in 8 of 10 compliant regulations (80.0%) compared with 16 of 28 non-compliant regulations (57.1%; p=0.269). Tobacco advertising restrictions (FCTC Article 13) appeared at broadly similar rates across compliance status (60.0% vs 53.6%; p=1.000), as did health education provisions (FCTC Article 12; 100.0% vs 96.4%; p=1.000) and ban on e-cigarette sales to minors (10.0% vs 21.4%; p=0.650). The observed patterns indicate that tobacco advertising restrictions and health education provisions appear at similar rates in compliant and non-compliant regulations, while cessation service and minor-protection provisions show higher frequencies in compliant regulations.

### Population coverage

The 10 jurisdictions classified as FCTC Article 8-compliant accounted for an estimated 121.6 million residents based on the 2020 Seventh National Population Census^17^, equivalent to approximately 33.0% of the population residing in the 38 jurisdictions covered by dedicated regulations and 8.6% of mainland China’s 2020 census population (1411.78 million). The 28 non-compliant jurisdictions accounted for an additional 246.7 million residents (17.5% of mainland China’s population). An estimated 1043 million residents (73.9% of mainland China’s population) reside in subnational jurisdictions not represented in the dedicated tobacco control corpus, some of whom may receive partial protection through embedded provisions in public health or civil-behavior regulations that fall outside the scope of this analysis. Supplementary Table S2 provides jurisdiction-level population estimates and a provincial-level supplementary matrix is presented in Supplementary file Table S1.

Of 31 provincial-level units in mainland China, 25 are represented by at least one dedicated regulation in the corpus, while 6 are not represented at the provincial or municipal level (Hainan, Hunan, Jiangsu, Jiangxi, Yunnan, and Tibet/Xizang). Absence from the dedicated corpus does not necessarily equate to absence of all legal protection, as some of these units may have smoke-free provisions embedded in public health or civil-behavior regulations not included in this analysis.

### Lanzhou: time-driven compliance loss as a cautionary case

Lanzhou is examined separately as a cautionary case illustrating how subnational compliance can be lost through administrative-rule expiration in the absence of substantive legislative action. The parent Lanzhou Municipal Regulation on Control of Smoking in Public Places (effective 1 January 2014; amended 30 March 2018 and 26 November 2021)^18^ retains Article 10, which expressly permits designated smoking rooms or smoking zones in restricted-smoking venues including restaurants, hotels, entertainment places, and airport interiors. The 2018 Lanzhou Municipal People’s Government Office Implementing Rules (effective 1 December 2018)^10^ superseded this permissive provision by declaring all indoor public places smoke-free from 1 January 2016 and prohibiting smoking rooms and smoking areas, thereby lifting Lanzhou to substantive Article 8 compliance. The Implementing Rules carried a five-year validity period and expired unrenewed on 30 November 2023. As of the analytical cutoff date (31 December 2024), no superseding normative document had been promulgated; the permissive Article 10 of the parent ordinance has reverted to controlling force, and Lanzhou no longer meets FCTC Article 8 compliance criteria under this study’s independent evaluation. The reliance on time-bounded administrative instruments rather than on primary legislation is an uncommon pathway in the corpus, but warrants attention for jurisdictions considering similar strategies.

## DISCUSSION

This systematic textual analysis of 38 Chinese subnational dedicated tobacco control regulations characterizes the textual features associated with FCTC Article 8 compliance. The findings are interpreted in light of the study’s exclusive focus on legal text; compliant drafting is a necessary but not sufficient condition for effective protection from secondhand smoke exposure, and the associations reported here do not establish causal relationships between text and outcome.

### Drafting scale and structural elaboration

Compliant regulations were associated with greater corpus scale and clause count, with medium effect sizes (Cliff’s δ=0.40 for character count and 0.46 for clause count) that reached the α=0.05 threshold for clause count (p=0.034) and approached it for character count (p=0.066). The consistency of these medium effects across both length measures, in the same direction and at similar magnitudes, lends interpretive weight to the pattern despite the borderline p-value for one of the two comparisons; with only 10 compliant regulations available, statistical power is limited and a sample of moderate-sized compliant regulations roughly twice the present size would be expected to confirm both differences at conventional thresholds. The pattern is consistent with the structural requirements of Article 8 implementation: effectively comprehensive coverage of indoor public places, workplaces, and public transport requires more extensive definitional and scope provisions; the absence of buffer periods and designated smoking areas requires explicit prohibition language; and effective enforcement requires detailed specification of authorities, procedures, and penalty schedules. The observation that the most compact compliant regulation in the corpus (Zhangjiakou, 1790 characters and 21 clauses) achieves compliance well below the median compliant length cautions against treating the observed medians as rigid normative thresholds. Compliant drafting at compact lengths is achievable when scope, exceptions, and enforcement are addressed concisely; further investigation of the drafting choices used by Zhangjiakou and similar compact compliant regulations would inform efficient drafting strategies. The findings are consistent with prior research by Yang et al.^8^ documenting the diversity of smoke-free legislation in mainland China and with the Lin et al.^5^ observation that adoption patterns are strongly shaped by jurisdiction-level legislative capacity. Comprehensive subnational smoke-free legislation in China has been independently associated with measurable reductions in smoking behavior and with declines in cardiovascular and cerebrovascular hospitalization, indicating that the textual features distinguishing compliant regulations are consequential for population health outcomes beyond the legal text itself^19,20^.

### The FCTC Article 8 compliance threshold

Only 10 of 38 regulations (26.3%) met FCTC Article 8 compliance criteria. This proportion is consistent with the China CDC Weekly inventory by Bi et al.^4^ which identified 46 cities achieving complete smoking bans across all eight WHO public-place categories when both dedicated and embedded instruments were considered. Non-compliance in this study’s dedicated corpus arose most commonly from exception clauses permitting designated smoking areas in hospitality venues, followed by enumerative listings of covered places that omitted relevant categories. For Chinese cities preparing dedicated tobacco control regulations, crossing the compliance threshold requires four concurrent drafting choices: adoption of effectively comprehensive coverage of the three core venue categories without exceptions for hospitality, entertainment, or workplace venues; removal of buffer periods or phased implementation; elimination of designated smoking rooms or smoking zones from the regulatory text; and explicit specification of enforcement authority and penalty schedules. The distribution of compliant regulations across all three policy eras indicates that the compliance threshold is achievable through deliberate drafting choices at any point in the legislative cycle and is not tied exclusively to any single political moment.

### E-cigarette prohibitions as a recent FCTC-aligned policy area

E-cigarette prohibitions appeared in 60.0% of compliant regulations compared with 25.0% of non-compliant regulations (p=0.062), an observed trend that did not reach the α=0.05 threshold but represents a meaningful gradient in absolute terms. As the FCTC framework has extended from combustible tobacco to electronic nicotine delivery systems through the 2016 COP7 report^11^ and the 2019 WHO guidance, and as China has issued its own e-cigarette administrative measures since November 2022^12^. Chinese cities drafting new legislation confront a choice between restricted and comprehensive product coverage. Integrated e-cigarette prohibitions offer an opportunity to align with recent FCTC-aligned policy areas while addressing a rapidly evolving product market; the absence of explicit e-cigarette provisions does not affect classification under the four core Article 8 criteria but represents a complementary drafting choice consistent with current evidence.

### Enforcement specification within compliance criteria

The Layer 4 comparison of individual and venue penalty specification and enforcement authority designation between compliant and non-compliant regulations characterizes how compliance criterion 4 is operationalized in practice rather than constituting an independent test of association: by construction, all 10 compliant regulations specify these components. The variability observed among non-compliant regulations (78.6–89.3%) indicates that several of these instruments specify some but not all components of criterion 4, suggesting partial-compliance pathways within the non-compliant category. The complaint and reporting hotline variable, which is not part of the compliance criteria, was present in 50.0% of compliant and 25.0% of non-compliant regulations (p=0.235); this represents an observed trend rather than a statistically supported difference. For non-compliant jurisdictions preparing amendments, completion of all components of criterion 4 is a prerequisite for moving toward compliance.

### Other FCTC complementary measures

Cessation service requirements (FCTC Article 14) appeared in 90.0% of compliant regulations compared with 50.0% of non-compliant regulations (p=0.056), an observed trend approaching the α=0.05 threshold. Bans on cigarette sales to minors (FCTC Article 16) appeared in 80.0% of compliant regulations compared with 57.1% of non-compliant regulations (p=0.269). Tobacco advertising restrictions (Article 13) and health education provisions (Article 12) appeared at broadly similar rates across compliance status, indicating that these measures have already diffused broadly across Chinese subnational tobacco control drafting. The consistently low adoption of e-cigarette sales restrictions to minors across the corpus (10.0% in compliant, 21.4% in non-compliant) points to a legislative gap that future amendments may productively address. These patterns are consistent with the global diffusion dynamics of FCTC implementation documented by Wipfli et al.^21^. The historical trajectory of smoke-free policy internationally shows that comprehensive coverage and complementary cessation provisions tend to consolidate together as jurisdictions mature toward full FCTC Article 8 alignment^22^.

### Population coverage and provincial representation

The 10 compliant jurisdictions cover an estimated 121.6 million residents (8.6% of mainland China’s 2020 population). Comparison with the 238.76 million coverage estimate reported by Bi et al.^4^ – which spans 46 cities across both dedicated and embedded instruments – illustrates the additional protection potentially afforded by public health, civil-behavior, and other broader regulatory instruments not included in the present analytical corpus. Six provincial-level units in mainland China are not represented at the dedicated level (Hainan, Hunan, Jiangsu, Jiangxi, Yunnan, and Tibet/Xizang); however, absence from the dedicated corpus should not be interpreted as absence of all legal protection, since alternative regulatory instruments may afford partial coverage in these units.

### The Lanzhou case and the architecture of sustainable compliance

The Lanzhou case illustrates a textual-architecture pathway in which compliance is achieved through supplementary administrative rules layered atop a permissive parent ordinance. When the 2018 Implementing Rules expired in November 2023 without renewal, Lanzhou reverted to the permissive parent text. This case is distinctive in the corpus: most compliant jurisdictions in the dataset achieved compliance through legislative amendment of the primary instrument, as illustrated by Xining’s 2021 amendment^23^ that deleted the permissive Articles 9, 10, and 12 of the 2014 original ordinance. For jurisdictions whose dedicated regulations contain permissive provisions, content-driven amendment of the primary legal instrument offers a structurally durable pathway not vulnerable to the time-limited validity that constrains administrative rules. Whether the Lanzhou strategy of ‘compliance through temporary administrative rules’ is used in other Chinese cities to avoid the more demanding People’s Congress legislative process, warrants further investigation.

### Implications beyond the Chinese subnational context

Although the findings are derived exclusively from Chinese subnational dedicated tobacco control regulations, the analytical logic developed here may hold relevance for other jurisdictions pursuing FCTC Article 8 implementation through subnational pathways in the absence of comprehensive national smoke-free legislation. The five-layer analytical framework, the attention to enforcement infrastructure as a component of compliance, the separation of e-cigarette prohibitions as a recent FCTC-aligned policy area, and the recognition that sustainable compliance is more durable when attained within primary legislative instruments rather than through supplementary administrative rules, are methodological contributions that do not depend on Chinese-specific legal institutions. Jurisdictions with similar legislative architectures may find the corpus-level triangulation of independent textual evaluation against authoritative expert classification a useful model for auditing Article 8 compliance in their own subnational legislative landscapes. Globally, WHO monitoring confirms that protection from tobacco smoke remains one of the less fully implemented FCTC measures, underscoring the broader relevance of drafting-architecture analysis for jurisdictions still pursuing complete smoke-free coverage^24^.

### Limitations

Several limitations should be considered when interpreting these findings. First, the corpus is restricted to dedicated tobacco control regulations and does not capture smoke-free provisions embedded within public health or civil-behavior regulations, which may afford partial protection in additional Chinese cities and provincial-level units. Second, this study evaluated textual architecture rather than implementation outcomes; compliant drafting is a necessary but not sufficient condition for effective protection from secondhand smoke exposure, and the broader determinants of effective tobacco control – governance capacity, enforcement context, health-system characteristics, and civil-society engagement – lie outside the scope of this corpus-level analysis. Third, the binary compliant/non-compliant classification simplifies a continuum of regulatory protection; partial and transitional compliance pathways are described in the granular coded variables but not captured in the headline classification. Fourth, the modest sample size of 10 compliant regulations limits statistical power for detecting differences in lower prevalence complementary measures; several comparisons approached but did not reach the α=0.05 threshold. Fifth, although inter-coder agreement was high (κ=0.94), residual subjectivity in coding decisions cannot be entirely excluded, particularly for the operationalization of ‘effectively comprehensive coverage’. Sixth, the analysis represents a snapshot at the 31 December 2024 analytical cutoff, and subsequent amendments – including any new legislation or implementing rules – are not captured. Seventh, the population coverage estimates use 2020 Seventh National Population Census figures and may not reflect post-2020 demographic change.

## CONCLUSIONS

This systematic textual analysis of 38 Chinese subnational dedicated tobacco control regulations characterizes the textual features associated with FCTC Article 8 compliance. Ten regulations (26.3%) met the four core requirements of the FCTC Article 8 Implementation Guidelines, covering an estimated 121.6 million residents (8.6% of mainland China’s 2020 population). Compared with non-compliant regulations, compliant regulations were associated with greater corpus scale and clause count, more complete specification of enforcement components, and higher frequencies of e-cigarette prohibitions and cessation service requirements. These observations are associations rather than causal claims and reflect features of legal text rather than implementation outcomes. For Chinese cities preparing or amending dedicated tobacco control regulations, the findings support three actionable drafting priorities: 1) adoption of effectively comprehensive coverage of indoor public places, workplaces, and public transport without exceptions for hospitality, entertainment, or workplace venues; 2) complete specification of enforcement authority, penalty schedules for both individuals and venues, and complaint-handling mechanisms within the primary legislative instrument; and 3) integration of contemporary FCTC-aligned measures including e-cigarette prohibitions and cessation service requirements. The analytical logic developed here is potentially extensible to other jurisdictions pursuing FCTC Article 8 implementation through subnational pathways.

## Supplementary Material



## Data Availability

The data supporting this research are available from the authors on reasonable request.
