# Supplementary material for: Textual analysis of 38 Chinese subnational tobacco control regulations against FCTC Article 8
Source: Tob Induc Dis. 2026 Jun 12;24:10.18332/tid/222367. doi: 10.18332/tid/222367 (PMC13261637; doi:10.18332/tid/222367)
Supplement: Supplementary file 1 [file TID-24-89-s1.pdf]

Supplementary Materials

Supplementary Figure S1. Document selection flowchart

Records identified through the National Database of Laws and Regulations, PKULaw, and provincial/municipal official websites (February–April 2025): approximately 240 records identified. Duplicates removed by exact title match across sources: approximately 130 records. Records screened against inclusion criteria: 110. Records excluded with reasons (embedded in patriotic health or civilized-behaviour regulation; not formally enacted; not at provincial or prefecture tier; not in force as of 31 December 2024): 72. Records included in final corpus: 38.

Supplementary Figure S1

Document selection pathway for 38 Chinese subnational dedicated tobacco control regulations
(enacted or materially amended after FCTC entry into force on 9 January 2006 through 31 December 2024)

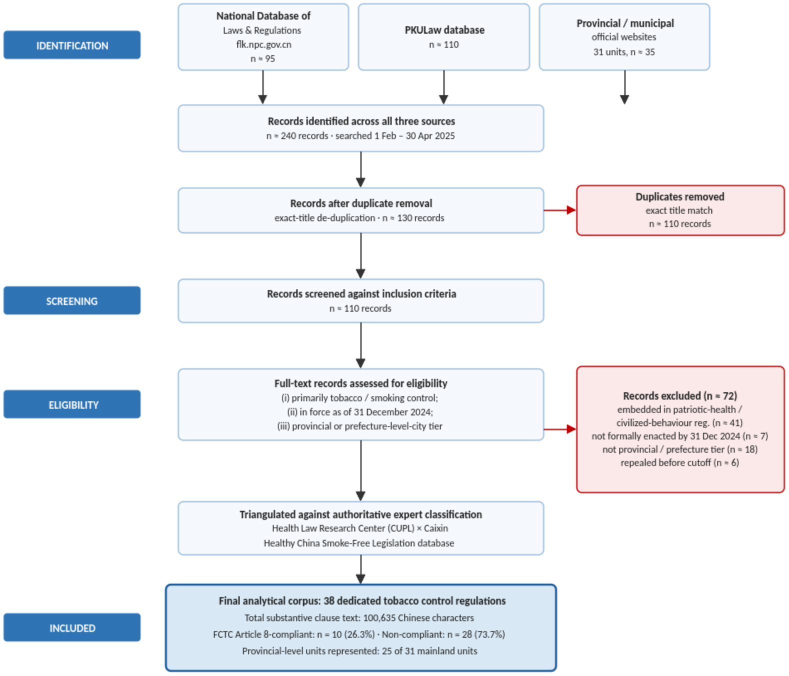

Embedded provisions in patriotic-health and civilized-behaviour regulations were excluded to preserve textual homogeneity for corpus-level comparison; this scope decision is acknowledged among the limitations. Record counts are approximate post-screening totals.

PCs = People's Congresses; PGs = People's Governments; CUPL = China University of Political Science and Law.

Supplementary Figure S2. Five-layer analytical framework

Layer 1 — Corpus scale and structure (character count, clause count). Layer 2 — FCTC Article 8 compliance classification (four core requirements). Layer 3 — Recent FCTC-aligned policy area (e-cigarette prohibitions). Layer 4 — Enforcement-related features (penalty schedules, enforcement authority — components of compliance criterion (iv); complaint hotline — independent variable). Layer 5 — Other FCTC complementary measures (Articles 12, 13, 14, 16). Inductively developed but anchored in the structure of the FCTC Article 8 Implementation Guidelines and the conventional architecture of dedicated tobacco control regulations.

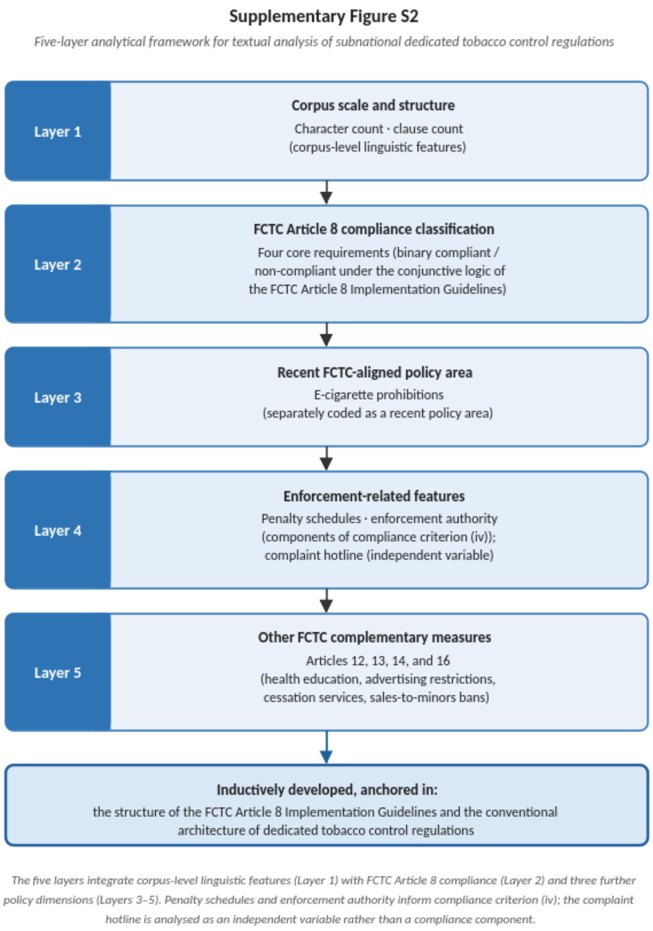

Supplementary Box S1. Worked examples of coding decisions for compliance criterion (i)

Compliant generic formulation. Example: Shenzhen SEZ Smoking Control Regulation (2014, as amended 2019), Article 4: “Smoking is prohibited in indoor public places, indoor workplaces, and public transport conveyances within the special economic zone.” This is a generic three-venue formulation, fully aligned with FCTC Article 8 implementation guidelines.

Compliant enumerative listing (substantively comprehensive). Example: A jurisdiction's regulation lists “government offices, schools, hospitals, libraries, museums, sports venues, hotels, restaurants, entertainment venues, shopping centres, transport waiting halls, and workplaces.” This enumerative list substantively covers the three core categories without exceptions for hospitality, entertainment, or workplace venues, and is therefore coded as compliant.

Non-compliant enumerative listing (with gaps). Example: A jurisdiction's regulation lists “government offices, schools, and hospitals” as smoke-free, with restaurants, hotels, and entertainment venues explicitly permitted to designate smoking areas. Although enumerative in form, the substantive coverage is incomplete, and the explicit exceptions for hospitality and entertainment venues render the provision non-compliant with FCTC Article 8 guidance.

Supplementary Table S1. Provincial-level coverage matrix for mainland China

All 31 mainland provincial-level units. Represented in dedicated corpus (with city or municipal-level regulation): 25 units — Beijing, Tianjin, Shanghai, Chongqing (4 direct-administered municipalities); Hebei (4 cities: Tangshan, Shijiazhuang, Qinhuangdao, Zhangjiakou); Henan (4: Anyang, Shangqiu, Luoyang, Zhengzhou); Shandong (4: Qingdao, Jinan, Weihai, Weifang); Guangdong (3: Zhuhai, Shenzhen, Guangzhou); Liaoning (3: Anshan, Shenyang, Dalian); and one city each in Anhui, Zhejiang, Fujian, Jilin, Guangxi, Inner Mongolia, Ningxia, Xinjiang, Gansu, Qinghai, Shanxi, Hubei, Shaanxi, Heilongjiang, Guizhou, and Sichuan. Not represented at the dedicated level: 6 units — Hainan, Hunan, Jiangsu, Jiangxi, Yunnan, and Tibet/Xizang. Absence from the dedicated corpus does not equate to absence of any legal protection: some unrepresented units may have smoke-free provisions embedded in patriotic health, civilized-behaviour, or other broader instruments not included in this analysis.

Supplementary Table S1. Provincial-level coverage matrix for 31 mainland Chinese provincial-level units

Companion to: Textual analysis of 38 Chinese subnational tobacco control regulations against FCTC Article 8 (corpus enacted or materially amended after FCTC entry into force on 9 January 2006 through 31 December 2024).

Provincial-level unit	Administrative category	Represented in dedicated corpus?	Cities in corpus (n)	Cities (English)	Compliant cities
Beijing	Municipality	Yes	1	Beijing	Beijing
Tianjin	Municipality	Yes	1	Tianjin	None
Shanghai	Municipality	Yes	1	Shanghai	Shanghai
Chongqing	Municipality	Yes	1	Chongqing	None
Hebei	Province	Yes	4	Tangshan, Shijiazhuang, Qinhuangdao, Zhangjiakou	Qinhuangdao, Zhangjiakou
Shanxi	Province	Yes	1	Datong	None
Liaoning	Province	Yes	3	Anshan, Shenyang, Dalian	None
Jilin	Province	Yes	1	Changchun	None
Heilongjiang	Province	Yes	1	Harbin	None
Jiangsu	Province	No	0	—	—
Zhejiang	Province	Yes	1	Hangzhou	Hangzhou
Anhui	Province	Yes	1	Suzhou (Anhui)	None
Fujian	Province	Yes	1	Fuzhou	None
Jiangxi	Province	No	0	—	—
Shandong	Province	Yes	4	Qingdao, Jinan, Weihai, Weifang	Qingdao
Henan	Province	Yes	4	Anyang, Shangqiu, Luoyang, Zhengzhou	None
Hubei	Province	Yes	1	Wuhan	Wuhan
Hunan	Province	No	0	—	—
Guangdong	Province	Yes	3	Zhuhai, Shenzhen, Guangzhou	Shenzhen
Hainan	Province	No	0	—	—
Sichuan	Province	Yes	1	Chengdu	None
Guizhou	Province	Yes	1	Guiyang	None
Yunnan	Province	No	0	—	—
Shaanxi	Province	Yes	1	Xi'an	Xi'an
Gansu	Province	Yes	1	Lanzhou	None
Qinghai	Province	Yes	1	Xining	Xining
Inner Mongolia	Autonomous Region	Yes	1	Hohhot	None
Guangxi	Autonomous Region	Yes	1	Nanning	None
Tibet / Xizang	Autonomous Region	No	0	—	—
Ningxia	Autonomous Region	Yes	1	Yinchuan	None
Xinjiang	Autonomous Region	Yes	1	Urumqi	None
**TOTAL**	**31 provincial-level units**	**25 of 31**	**38**	**38 cities/jurisdictions in corpus**	**10 compliant cities (26.3%)**

Legend

Blue fill	Provincial-level unit represented by at least one FCTC Article 8-compliant city in the corpus.
Yellow fill	Provincial-level unit represented in the corpus but no constituent city met FCTC Article 8 compliance criteria.
Red fill	Provincial-level unit not represented at the dedicated level (no dedicated tobacco control regulation in the analytical corpus).

Notes.

(1) Absence from the dedicated corpus does not equate to absence of all legal protection. Some unrepresented provincial-level units (e.g., Jiangsu, Hunan, Yunnan) may have smoke-free provisions embedded in patriotic-health (爱国卫生条例) or civilized-behaviour (文明行为条例) regulations, which fall outside the scope of this corpus-level textual analysis.

(2) Hong Kong SAR, Macau SAR, and Taiwan are excluded; the 31-unit denominator covers mainland China only.

(3) Suzhou (Anhui) refers to 宿州 in Anhui Province, distinct from 苏州 in Jiangsu Province.

(4) Compliance evaluation follows the four core requirements of the FCTC Article 8 Implementation Guidelines (FCTC/COP2(7), 2007) and was triangulated against the Health Law Research Center (CUPL) × Caixin Data Visualization Lab authoritative classification.

Supplementary Table S2. Population coverage estimates by jurisdiction (2020 Seventh National Population Census)

Compliant jurisdictions (n = 10; total population 121.6 million; 8.6% of mainland China population): Shanghai 24.87 M; Beijing 21.89 M; Shenzhen 17.56 M; Xi'an 12.95 M; Wuhan 12.33 M; Hangzhou 11.94 M; Qingdao 10.07 M; Zhangjiakou 4.42 M; Qinhuangdao 3.14 M; Xining 2.47 M. Non-compliant jurisdictions (n = 28; total population 246.7 million; 17.5% of mainland China population): Chongqing 32.05 M; Chengdu 20.94 M; Guangzhou 18.68 M; Tianjin 13.87 M; Zhengzhou 12.60 M; Shijiazhuang 11.23 M; Harbin 10.01 M; Weifang 9.39 M; Jinan 9.20 M; Changchun 9.07 M; Shenyang 9.07 M; Nanning 8.74 M; Fuzhou 8.29 M; Anyang 5.48 M; Shangqiu 7.72 M; Tangshan 7.72 M; Dalian 7.45 M; Luoyang 7.06 M; Guiyang 5.99 M; Suzhou (Anhui) 5.32 M; Lanzhou 4.36 M; Urumqi 4.05 M; Anshan 3.65 M; Hohhot 3.45 M; Datong 3.10 M; Weihai 2.91 M; Yinchuan 2.86 M; Zhuhai 2.44 M. Mainland China total (2020 Census): 1,411.78 million. Population not represented in dedicated corpus (estimated): 1,043 million (73.9% of mainland China).

Supplementary Table S2. Population coverage estimates by jurisdiction (2020 Seve

Companion to: Textual analysis of 38 Chinese subnational tobacco control regulations against FCTC Article 8. Population figures are resident population from the 2020 Seventh National Population Census; the mainland China denominator is 1,411.78 million.

ID	City / jurisdiction	Province	FCTC Art. 8 compliant	2020 population (million)
**FCTC Article 8-compliant jurisdictions (n = 10)**				
38	Shanghai	Shanghai	Yes	24,87
20	Beijing	Beijing	Yes	21,89
6	Shenzhen	Guangdong	Yes	17,56
31	Xi'an	Shaanxi	Yes	12,95
29	Wuhan	Hubei	Yes	12,33
5	Hangzhou	Zhejiang	Yes	11,94
11	Qingdao	Shandong	Yes	10,07
33	Zhangjiakou	Hebei	Yes	4,42
26	Qinhuangdao	Hebei	Yes	3,14
25	Xining	Qinghai	Yes	2,47
			**Compliant subtotal**	**121,64**
= 33.0% of the 38-jurisdiction dedicated corpus population; 8.6% of mainland China's 2020 census population				
**FCTC Article 8 non-compliant jurisdictions (n = 28)**				
21	Chongqing	Chongqing	No	32,05
35	Chengdu	Sichuan	No	20,94
32	Guangzhou	Guangdong	No	18,68
13	Tianjin	Tianjin	No	13,87
28	Zhengzhou	Henan	No	12,6
14	Shijiazhuang	Hebei	No	11,23
30	Harbin	Heilongjiang	No	10,01
37	Weifang	Shandong	No	9,39
15	Jinan	Shandong	No	9,2
8	Changchun	Jilin	No	9,07
23	Shenyang	Liaoning	No	9,07
9	Nanning	Guangxi	No	8,74
7	Fuzhou	Fujian	No	8,29
2	Shangqiu	Henan	No	7,72
10	Tangshan	Hebei	No	7,72
24	Dalian	Liaoning	No	7,45
19	Luoyang	Henan	No	7,06
34	Guiyang	Guizhou	No	5,99
1	Anyang	Henan	No	5,48
3	Suzhou (Anhui)	Anhui	No	5,32
22	Lanzhou	Gansu	No	4,36
18	Urumqi	Xinjiang	No	4,05
12	Anshan	Liaoning	No	3,65
16	Hohhot	Inner Mongolia	No	3,45
27	Datong	Shanxi	No	3,1
36	Weihai	Shandong	No	2,91
17	Yinchuan	Ningxia	No	2,86
4	Zhuhai	Guangdong	No	2,44
			**Non-compliant subtotal**	**246,7**
= 67.0% of the 38-jurisdiction dedicated corpus population; 17.5% of mainland China's 2020 census population				
**Corpus and national totals**				
			**Total population, 38-jurisdiction dedicated corpus**	**368,34**
26.1% of mainland China's 2020 census population				
			**Mainland China total (2020 Seventh National Population Census)**	**1411,78**
denominator				
			**Population outside the dedicated corpus (estimated)**	**1043,44**
73.9% of mainland China; may have partial protection via embedded provisions outside this corpus				

Notes.

(1) Population figures are resident population (常住人口) from the 2020 Seventh National Population Census, in millions, rounded to two decimals.

(2) The mainland China denominator (1,411.78 million) excludes Hong Kong SAR, Macau SAR, and Taiwan.

(3) For directly administered municipalities (Beijing, Tianjin, Shanghai, Chongqing) the figure is the whole-municipality population; for prefecture-level cities it is the whole-city (municipal) population, consistent with the

(4) The percentages are arithmetic shares; they do not adjust for within-jurisdiction implementation or enforcement, which lie outside the textual scope of this study (see Limitations).

(5) Suzhou (Anhui) refers to 宿州 in Anhui Province, distinct from 苏州 in Jiangsu Province.

© 2026 Chen Y. et al.
